# Supplementary material for: Diversity in Proprotein Convertase Reactivity among Human Papillomavirus Types
Source: Viruses. 2023 Dec 26;16(1):39. doi: 10.3390/v16010039 (PMC10820984; doi:10.3390/v16010039)
Supplement: Supplementary file 1 [file viruses-16-00039-s001.zip › viruses-2761404-supplementary.pdf]

viruses

MDPI

Supplement

1. Materials and Methods

Production of native HPV particles and infectivity assays

Native HPV16 virions were produced and tested as described before [28]. Linear wild-type HPV16 (114/B) genome was electroporated into cervical keratinocytes obtained from cervical biopsies and immortalized by stably maintaining episomal HPV16. To produce native HPV virions, HPV-containing keratinocytes were grown in raft cultures, and mature virus particles were harvested after 20-days. HPV positive rafts were harvested, and virus titers determined using a qPCR-based DNA encapsidation assay against a standard curve using purified viral genomes. Assays for the infection of HaCat cells were performed using a RT-qPCR-based method that measures levels of E1^E4 early viral transcripts.

Activation of native virions with PCs

Virus dilutions were treated with PCs as described in the Materials and Methods section for the activation of PsV particles, and activation was measured by infectivity assays. Infectivity assay values and their standard deviation of virus treated with several PCs are shown in Figure S3.

Quantification of PC gene expression in HEK293TT cells

The levels of PC gene transcript expression in the cell line used for HPV PsV cell entry assays was performed using the same methodology and set of primers used for the quantification of PC gene expression in keratinocytes from anatomic sites and described in the Materials and Methods section. The transcription levels and their standard deviation values are presented in Table S2.

Determination of cell viability

HEK293TT cells were grown adherent on collagen coated 96-well plates and treated with 500 nM *a*1PDX or CMK for 2h or 24h. Cell viability was measured using the CellTiter-Glo Luminescent Cell viability Assay reagent (Promega, Madison, WI, USA). Luminescence values were taken after 10 min incubation. Percentage values with respect to control cells that were not treated with inhibitors and their standard errors are presented in Table S5.

2. Figures and Tables

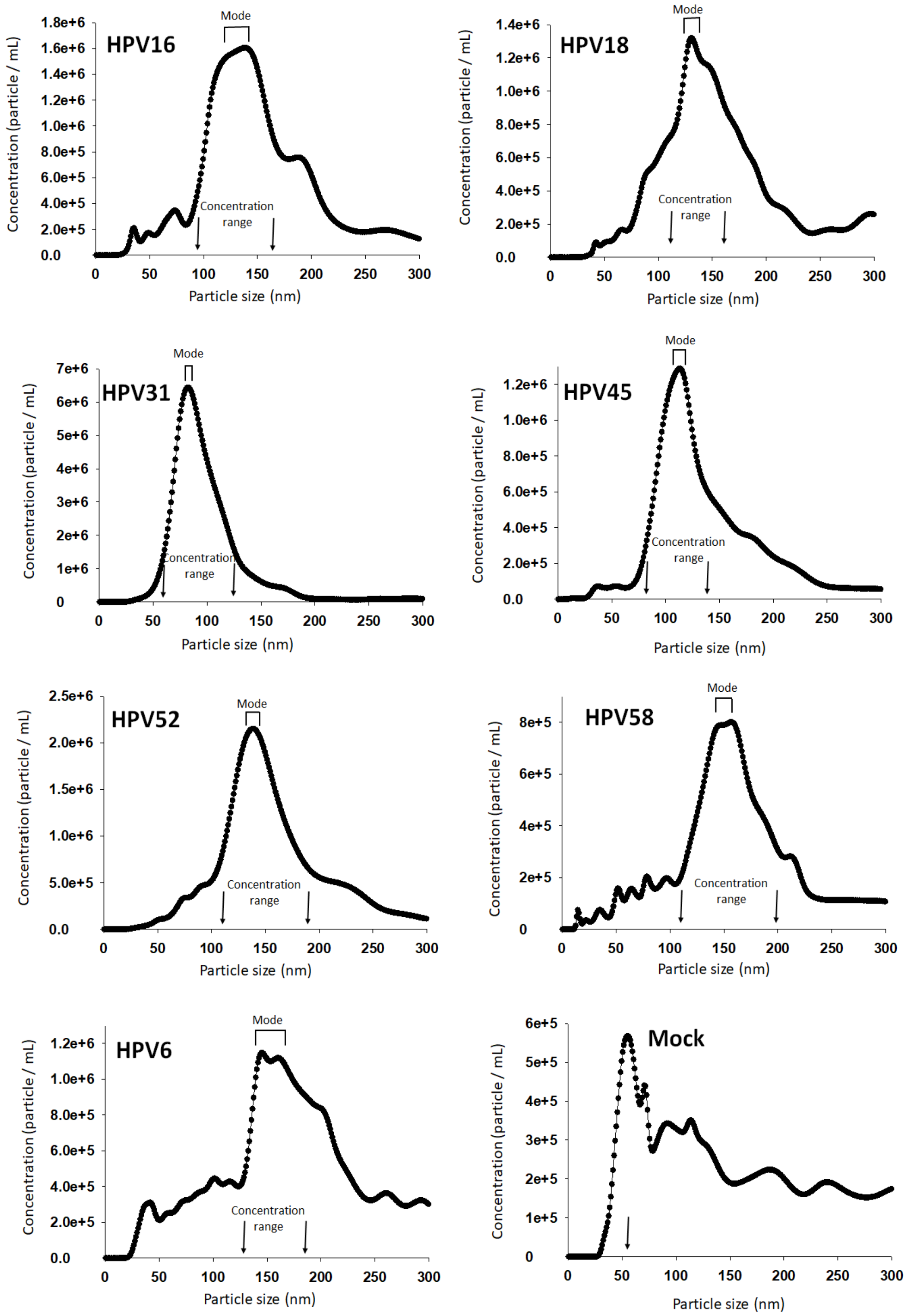

Figure S1. *PsV concentration and size distribution*. Particle concentrations were determined by nanoparticle tracking analysis (NanoSight 300, Malvern Panalytical). Gel filtration purified PsV particles were analyzed. Each profile is the average of 9-15 runs. The area under the distribution curve between the two arrows was estimated as the particle concentration. The Mode range corresponds to the particle size with the highest concentration. Concentration values are listed in Table S3. The mock plot shows the analysis of a PsV non-containing sample similarly treated as the others.

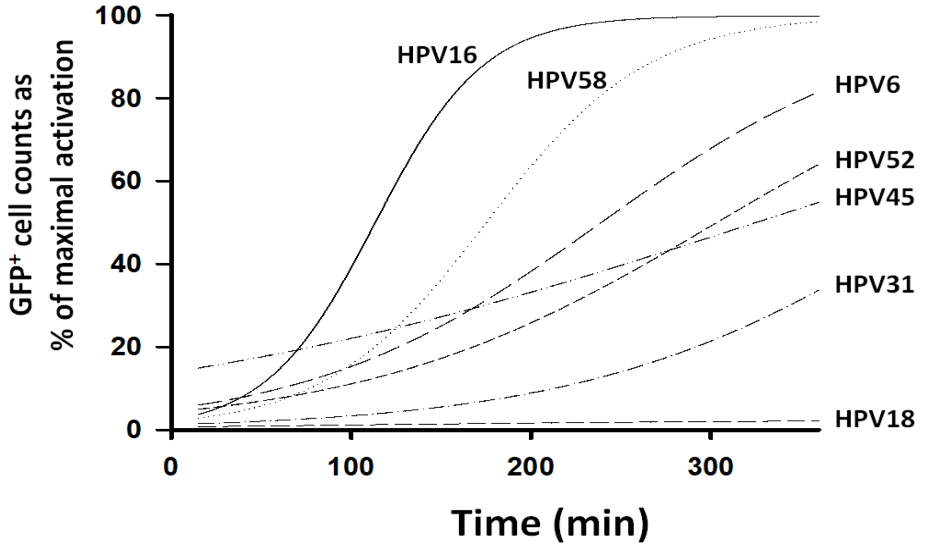

Figure S2. *Computer fitting of time-dependent activation curves.* HPV types were activated with furin overtime (Figure 3B). The experimental activation curves were fitted to the sigmoidal equation (Sigma Plot software). Inflection points (Table S4) provided a measure of the differences in reactivity.

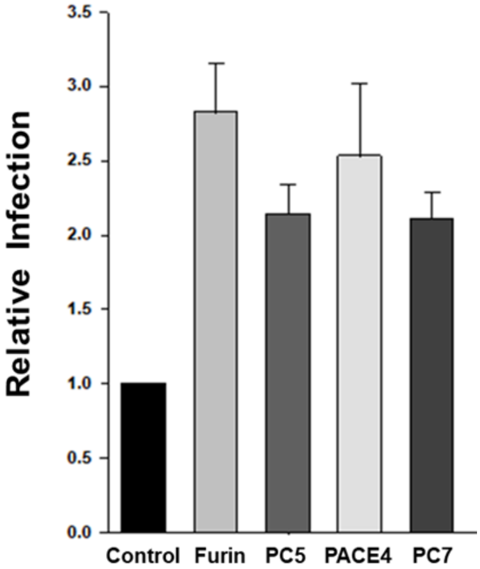

Figure S3. *Activation of native HPV16 with PCs*. HaCat cells were infected with native HPV16, and infectivity was measured by qPRC two days post infection. Before infection, virus was incubated without (control) of with 40 nM of a PC at 37°C overnight. Data represents the average and standard deviation from one experiment in triplicate.

Table S1. *Expression of PC genes in human keratinocytes grown in rafts from primary cells*. PC gene expression and their standard deviation values were obtained from four cell cultures analyzed separately and each determination was done in triplicates. These are raw PC and GAPDH gene expression values standardized to the cDNA concentration. The percentage of the total PC gene expression with respect to the GAPDH expression for each site was plotted in Figure 1A. The percentage of the expression of each PC with respect to the total in each site was plotted in Figure 1B.

	CERVIX	ANUS	GINGIVA	TONSILS	FORESKIN	Abundance
**FURIN**	305.0 ± 103.6	722.7 ± 113.0	346.1 ± 158.4	215.8 ± 43.2	684.9 ± 143.8	2275
**PC4**	182.6 ± 52.7	470.4 ± 31.5	172.1 ± 93.5	64.48 ± 21.60	485.3 ± 128.7	1375
**PC5**	21.95 ± 6.68	51.39 ± 13.36	82.5 ± 41.4	23.96 ± 4.94	30.36 ± 11.17	210
**PACE4**	176.1 ± 45.5	498.2 ± 79.0	170.6 ± 85.4	66.40 ± 16.99	496.8 ± 147.6	1408
**PC7**	60.97 ± 20.81	101.2 ± 10.9	85.71 ± 33.53	69.68 ± 16.32	87.10 ± 22.06	405
**GAPDH**	10600 ± 330	10110 ± 1240	11770 ± 5270	10690 ± 3370	10520 ± 2710	
**PC total**	746.6	1844	857.0	440.3	1784	
**FURIN/total (%)**	41	39	40	49	38	

Table S2. *Expression of PC genes in HEK293TT cells.* Gene expression values were standardized to the cDNA concentration and proportionally to the expression of the GAPDH gene adjusted to a value of 10,000. Expression value averages and their standard deviation were determined from one experiment in triplicate.

	Gene expression	Individual PC gene expression as % of total PC gene expression
**Furin**	274 ± 0.7	30.0
**PC4**	354 ± 3	38.8
**PC5**	51 ± 0.1	5.6
**PACE4**	190 ± 2	20.8
**PC7**	43 ± 0.3	4.7
**Total PC gene expression as % of GAPDH**	9.1	---------

Table S3. *PsV nanoparticle tracking analysis. Particle concentrations were determined as described in Figure S1.* Concentration values are shown with their associated standard deviation values. The PsV particles for each HPV type were titrated into the entry assay to determine the concentration range for each type that produced the appropriate entry values to measure activation with PCs and inhibition with PC inhibitors. Entry values with PC-untreated PsV particles, in which entry depends on the cell PC activity, or pre-activated with 5 nM furin for 3 h at 37 °C, in which the cell PC activity is inhibited with CMK, are shown here and plotted in Figures 2 and 3, respectively.

HPV	Concentration (Particles / mL)	Entry values #GFP+ cell counts / 10^4 particles		Furin / Cell PCs activation ratio
		Untreated PsV No CMK	Furin treated PsV CMK-treated cells	
**6**	$(3.6 \pm 1.9) \times 10^6$	19 ± 7	112 ± 28	5.9
**16**	$(3.0 \pm 1.9) \times 10^7$	174 ± 63	221 ± 76	1.3
**18**	$(1.3 \pm 0.6) \times 10^7$	31 ± 14	31 ± 14	1
**31**	$(1.4 \pm 0.3) \times 10^8$	9 ± 2	10 ± 3	1.1
**45**	$(7.5 \pm 1.1) \times 10^6$	134 ± 36	183 ± 35	1.4
**52**	$(2.7 \pm 0.2) \times 10^7$	27 ± 8	60 ± 10	2.2

Table S4. *Inflection point for the time-dependent curves of HPV preactivation with furin***.** The experimental preactivation curves shown in Figure 3B were fitted to the sigmoidal equation by linear regression analysis (Figure S2). Computer fittings using the program Sigma Plot were made having the amplitude fixed to 100 %. The calculated error is expressed as the coefficient of variance (CV).

HPV	Inflection Point (min)	CV (%)	Regression coefficient (R)
**6**	238	5.0	0.978
**16**	113	0.67	1.0
**18**	1800	24	0.776
**31**	426	1.4	0.998
**45**	325	2.1	0.994
**52**	303	3.3	0.984
**58**	175	1.0	0.999

Table S5. *Viability of HEK293TT cells treated with α1PDX or CMK*. HEK293TT cells were exposed to 500 nM PC inhibitor and then tested for viability. Each data value represents average and standard deviation from one experiment done in triplicate.

	**Cell viability (%)**	
	**2 h treatment**	**24 h treatment**
**Control**	100 ± 8	100 ± 10
**α1PDX**	93 ± 5	94 ± 8
**CMK**	92 ± 7	100 ± 5
